# Plasma KL-6 as a Potential Biomarker for Bronchopulmonary Dysplasia in Preterm Infants

**DOI:** 10.1155/2024/3623948

**Published:** 2024-01-30

**Authors:** Petya Radulova, Margaritka Boncheva, Gencho Nachev, Boriana Slancheva, Violeta Dimitrova

**Affiliations:** ^1^Department of Obstetrics and Gynecology, Medical University of Sofia, Zdrave 2 Street, Sofia 1431, Bulgaria; ^2^Neonatology Clinic, University Hospital of Obstetrics and Gynecology “Maichin Dom”, Sofia, Bulgaria; ^3^University Hospital for Active Treatment “St. Ekaterina”, Pencho Slaveikov Street, 52A, Sofia 1431, Bulgaria

## Abstract

**Background:**

KL-6 is a biomarker of interstitial lung injury and increases during repair.

**Aim:**

Our aim was to determine the predictive value of plasma KL-6 for the development of bronchopulmonary dysplasia (BPD) in preterm infants.

**Methods:**

Ninety-five extremely preterm infants (EPIs), born at <28 gestational age (GA), were divided into two main BPD groups as follows: the moderate/severe and the no/mild group. KL-6 was analyzed on days 7 and 14. Binary logistic regression analyses and ROC curve analyses were performed.

**Results:**

Infants <26 + 0 weeks' GA have higher mean KL-6 than infants >25 + 6 weeks' GA on 7 and 14 days (335 vs. 286 U/ml and 378 vs. 260 U/ml; *p* = 0.005 and 0.018, respectively). In the binary regression model at KL-6 day 7, three of the prognostic factors remained significant—mechanical ventilation OR: 10.38 (95% CI: 3.57–30.14), PDA OR: 6.39 (95% CI: 0.87–46.74), and KL-6 OR: 4.98 (95% CI: 1.54–16.08). The AUC was 0.86 with a sensitivity and specificity of 79% at a cutoff value ≥0.34. In the binary regression model at KL-6 day 14, six of the prognostic factors were significant—PDA OR: 23.34 (95% CI: 2.14–254.24), KL-6 OR: 13.59 (95% CI: 3.19–57.96), GA OR: 4.58 (95% CI: 1.16–18.06), mechanical ventilation OR: 4.45 (95% CI: 1.23–16.16), antenatal steroids OR: 0.19 (95% CI: 0.04–0.95), and gender (female OR: 0.30 (95% CI 0.08–1.12)). The AUC was 0.91, and the sensitivity and accuracy for a cutoff ≥0.37 were 89% and 85%, respectively.

**Conclusion:**

KL-6 could be a useful screening biomarker for early detection of infants at increased risk for developing BPD.

## 1. Introduction

Bronchopulmonary dysplasia (BPD) is a chronic inflammatory lung disease that occurs mostly in extremely preterm infants (EPIs) [1]. In recent years, the incidence of this disease is high, mainly because a higher percentage of preterm infants survived [2]. BPD is a complex and multifactorial disease, with many prenatal, perinatal, and postnatal factors (grade of prematurity, oxygen toxicity, mechanical ventilation, infections, chorioamnionitis, and preeclampsia) contributing to its development [3].

Numerous clinical studies have emphasized that lung inflammation is the main cause of BPD [4, 5]. Plasma proteins such as basal cell adhesion molecule (BCAM), sialic acid-binding Ig-like lectin 14 (SIGLEC-14), N-terminal propeptide of type 3 collagen, and SP-A and anti-SP-A immune complexes can serve as biomarkers for early detection of the disease [6, 7]. However, most of these tests are not lung specific and the laboratory tests used are complicated. Krebs von den Lungen (KL-6) is a glycoprotein mainly expressed and secreted by bronchial epithelial cells and pneumocytes type II, and its expression correlates with the presence and severity of various chronic lung diseases. KL-6 is also associated with pulmonary fibrosis [8].

Numerous studies demonstrate that KL-6 plasma levels are elevated in patients with various types of interstitial pneumonia, for which fibrosis and type 2 alveolar hyperplasia are typical. In contrast, plasma levels of KL-6 are not elevated in many noninterstitial lung diseases such as bronchial asthma, bacterial pneumonia, and emphysema [9, 10]. In preterm infants with BPD, the pathological lung changes are very similar to those observed in the lungs of patients with interstitial pneumonia [11, 12]. In small cohort of 42 infants, Ogihara et al. reported that KL-6 plasma levels at weeks 1 and 2 seem to be good predictors of moderate/severe BPD in patients <28 weeks GA (positive predictive values of 83% and 80%, respectively) [13].

A recently published online clinical tool estimated the risk of BPD development in EPI based on the predictive value of different clinical data [14]. The results showed that among 9181 included infants, birth weight was the most predictive factor for death or BPD severity on postnatal day 1, while the mode of respiratory support was the most predictive factor on days 3, 7, 14, and 28 [14]. The predictive accuracy of the models increased at each time period from postnatal day 1 (C-statistic: 0.674) to postnatal day 28 (C-statistic: 0.741).

We decided to make a risk prediction model of BPD development like the online clinical tool [14], including KL-6 plasma levels on day 7 and 14, based on the findings by Ogihara et al. [13]. The aim was to evaluate the potential of KL-6 as a biomarker in routine clinical practice as early as the first two weeks of life and to improve the current risk stratification for the development of BPD in EPIs less than 28 GA.

## 2. Materials and Methods

### 2.1. Study Design and Subjects

This study was designed as a prospective single-center study. Over a 3-year period (between June 2020 and May 2023), 95 EPIs with a gestational age of less than 28 weeks' GA were enrolled. We made a subanalysis of KL-6 in infants with GA < 26 + 0 weeks, as these infants have a highest probability of severe BPD development in our department. The study protocol was reviewed and approved by our institutional review board and conducted in accordance with good clinical practice guidelines. Written informed consent was obtained from all parents. Inclusion criteria were a gestational age of <28 weeks and the absence of major congenital malformations. Plasma levels KL-6 (U/ml) were analyzed on the 7th and 14th day of life (DOL) and the values were described in special tables. The laboratory method used was the CLEIA FujiRebio LUMIPULSE G600II KL-6.

BPD was defined as oxygen dependence for at least 28 days, and severity was classified as mild (21% oxygen), moderate (21% to 30% oxygen), and severe (>30% and/or positive pressure support) at week 36 GA [12]. This is an old classification of BPD, but in our institution, we used it during the study period. Based on these criteria, patients were divided into two groups as follows: the group without or mild BPD (48 babies) and the group with moderate-severe BPD (47 babies).

### 2.2. Blood Collection and Laboratory Technique

Venous blood (0.5 ml) was collected from the infants on the 7th and 14th DOL. The heparinized blood samples were centrifuged at 2800 × *g* for 8–10 min at 4°C to collect plasma. KL-6 was measured using a colorimetric quantitative sandwich enzyme-linked immunosorbent assay kit (CLEIA FujiRebio LUMIPULSE G600II KL-6). KL-6 was measured in U/ml, with a sensitivity of only 1.12 U/ml.

### 2.3. Statistical Analysis

Statistical analysis was performed using IBM SPSS Statistics 25.0. A two-tailed *P* value of less than 0.05 was considered to indicate a statistically significant difference. The following methods were used: Fisher's exact test, test of Kolmogorov–Smirnov and Shapiro–Wilk, two-independent samples test of Mann–Whitney U, correlation analysis, binary logistic regression analysis, ROC curve analysis, and screening tests validation criteria. To quantitatively assess the risk of developing moderate and severe BPD, we made a binary logistic correlation analysis. To assess the combined influence of the investigated risk factors, we put all of them and started the procedure “backward conditional.”

## 3. Results

The main perinatal characteristics of the patients in each group are shown in Table 1.

The two groups studied differ significantly in all quantitative variables. Infants with moderate/severe BPD had higher mean values for mechanical ventilation, O2 therapy, and hospital stay and lower scores for weight and GA. For categorical variables, a statistically significant difference was found for antenatal steroids, PDA (lung hemorrhage), and ROP. With the exception of prenatal steroids, infants with moderate/severe BPD had greater relative proportions for the other two variables.

**Figure 1:**
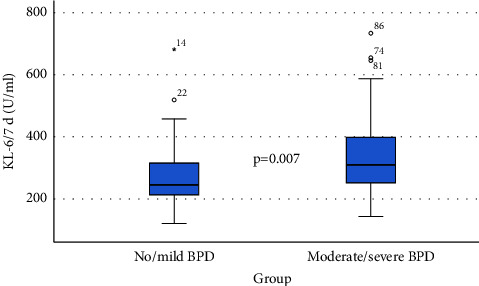
Box plot of KL-6 at day 7 for both groups of investigation.

**Figure 2:**
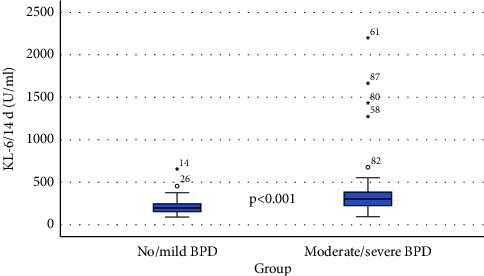
Box plot of KL-6 at day 14 for both groups of investigation.

Figures 1 and 2 show box plots of the values of the investigated biomarker in the study groups at both measurement time points. They showed that the ability to distinguish infants with no/mild BPD from those with moderate/severe BPD using the biomarker KL-6 was higher at day 14.

We made an analysis of the dependence between the investigated biomarker and the following characteristics: GA, asphyxia, complicated pregnancy (eclampsia, preeclampsia, chorioamnionitis, diabetes mellitus type 1 and 2, and gestational diabetes), NEC and/or sepsis, and mechanical ventilation. The results were as follows:

- Infants <26 + 0 weeks' GA have higher mean KL-6 than infants with GA > 25 + 6 weeks on 7 and 14 days (335 vs. 286 U/ml and 378 vs. 260 U/ml; *P*=0.005 and 0.018, respectively)
- Mechanical ventilation: a weak positive correlation was found, meaning that infants with higher biomarker values were mechanically ventilated longer (p: 0.043 for KL-6 on 7 day and p: 0.015 for KL-6 on 14 day)
- A statistically significant dependence between the values of the tested biomarker and the indicators asphyxia, complicated pregnancy, NEC, and/or sepsis and was not substantiated

**Figure 3:**
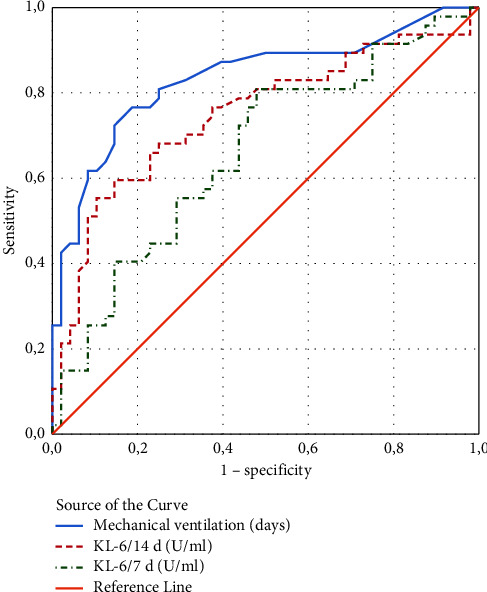
ROC curve of plasma KL-6 and mechanical ventilation (days) to distinguish infants with no/mild BPD from those with moderate/severe BPD.

**Figure 4:**
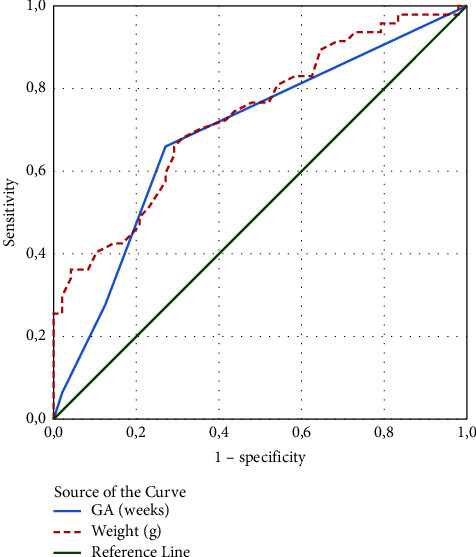
ROC curve of GA (weeks) and weight to distinguish infants with no/mild BPD from those with moderate/severe BPD.

We tested the following indicators as potential risk factors for moderate/severe BPD: KL-6 on day 7, KL-6 on day 14, PDA (lung hemorrhage), mechanical ventilation (days), GA (weeks), weight (g), antenatal steroids, gender, C-section, complicated pregnancy, and Apgar score <3 at 1 minute. Because the variables KL-6, mechanical ventilation, GA, and weight are quantitative variables, a ROC curve analysis was used to establish a cutoff value to convert them to qualitative variables. The Youden index (maximum (sensitivity + specificity − 1)) was used in the selection of the cutoff value (Figures 3 and 4 and Table 2).

With the established threshold value of KL-6 on day 7, we can achieve very good sensitivity and satisfactory specificity, while with KL-6 on day 14, we can achieve very good specificity and good sensitivity (Table 2).

To quantitatively assess the risk of obtaining moderate/severe BPD, we performed a binary logistic correlation analysis.

### 3.1. Binary Regression Model at KL-6/7d

We found that for 6 of the 10 prognostic factors studied (Table 3), the crude OR was significant; the 6 factors are KL-6/7d, PDA (lung hemorrhage), mechanical ventilation, GA, weight, and antenatal steroids. Mechanical ventilation has the highest OR (14.18), followed by PDA (OR = 8.79) and KL-6/7d (OR = 4.59). Only antenatal steroids have a protective influence (about 66%). To evaluate the combined influence of the studied characteristics, we put them all together and started the “backward conditional” procedure. Finally, three of the prognostic factors remained in the equation, mechanical ventilation with the highest OR (10.38), PDA (OR = 6.39), and KL-6/7d (OR = 4.98).

The result was the following binary logistic equation:

(1) $$\begin{array}{c} P=\frac{1}{1+e^{-z}}, \end{array}$$

where **Z** = **1.605KL-6/7d** + **1.854PDA** + **2.339** **MV** − **2.385**.

**Figure 5:**
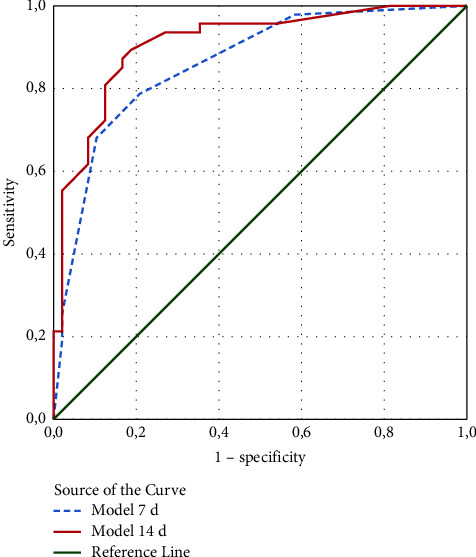
ROC curve of model 7d and model 14d to distinguish infants with no/mild BPD from those with moderate/severe BPD.

For this equation, the AUC = 0.86 (Figure 5) and the sensitivity and accuracy for the cutoff ≥0.34 are 79% (Table 2). The values of the variables are 1 if they are on the corresponding side of the threshold and “yes” for “PDA.” Otherwise, they are zeros.

### 3.2. Binary Regression Model at KL-6/14d

In order to evaluate the combined influence of the studied characteristics, we also used here all the studied characteristics and started the “backward conditional” procedure. Finally, 6 of the prognostic factors (Table 4) remained in the equation, PDA with the highest OR (23.34), KL-6/14d (OR = 13.59), gestational age (OR = 4.58), mechanical ventilation (OR = 4.45), antenatal steroids (OR = 0.19), and sex (female OR = 0.30).

The result was as follows:

(2) $$\begin{array}{c} Z=\frac{2.610\text{ KL}-6}{14\;d\;}+3.150\text{ PDA}+1.494\text{ MV}+1.522\text{ GA }–1.664\text{ antenatal steroids }–1.189\text{ Sex}+0.415. \end{array}$$

For this equation, the AUC = 0.91 (Figure 5) and sensitivity and accuracy for cutoff ≥0.37 are 89% and 85%, respectively (Table 2).

## 4. Discussion

BPD is a multifactorial disease with numerous prenatal, neonatal, and genetic factors contributing to its development. Known risk factors include maternal chorioamnionitis, intrauterine growth retardation, low gestational age and birth weight, prolonged mechanical ventilation, persistent ductus arteriosus (PDA) and pulmonary hemorrhage, perinatal and postnatal infections, and prolonged oxygen therapy [1, 15].

In the present study, the main clinical data of 95 infants were recorded, including the general characteristics of the infants, maternal characteristics, duration of mechanical ventilation, oxygen therapy, neonatal morbidities, duration of hospitalization, and KL-6 on days 7 and 14.

KL-6 is a known biomarker of lung injury that increases when repair processes begin. Wang et al. reported levels no higher than 200 U/ml, whereas Ogihara et al. reported that KL-6 plasma levels of 199 U/ml at week 1 or 232 U/ml at week 2 are excellent predictors of moderate/severe BPD in patients <28 weeks GA (positive predictive values of 83% and 80%, respectively) [13, 16]. Our results show a statistically significant difference in the values of KL-6/7d and KL-6/14d between infants with GA ≤ 26 weeks (mean KL-6/7d–335.73 U/ml and mean KL-6/14d–378.34 U/ml) and infants with GA > 26 weeks (mean KL-6/7d–286.92 U/ml and mean KL-6/14d–260.64 U/ml) with *P* values of 0.005/7d and 0.018/14d, respectively. A weak positive correlation was found between the days of mechanical ventilation and the values of KL-6 at both measurement time points (*P* 0.043 and *P* 0.015, respectively). No statistically significant dependence was found between the values of the tested biomarker and the indicators asphyxia, complicated pregnancy, NEC, and/or sepsis.

We tested several indicators as potential risk factors for moderate/severe BPD; KL-6/7d, KL-6/14d, PDA (lung hemorrhage), mechanical ventilation (days), GA (weeks), weight (g), antenatal steroids, sex, C-section, complicated pregnancy, and Apgar score <3 at 1 minute, and ROC curve analysis was used to set a cutoff value. The results show that with the established threshold of KL-6/7d (≥247.5), we can achieve a very good sensitivity of 81% and a satisfactory specificity of 52%, whereas with KL-6/14d (≥277.5), we can achieve a very good specificity of 85% and a good sensitivity of 60%.

Because of the unsatisfactory values for specificity and sensitivity of the biomarker tested, we decided to perform binary logistic correlation analysis, and early predictive models for BPD 7d and 14d were developed based on clinical data. In the binary regression model on KL-6/7d, three of the prognostic factors remained in the equation, with mechanical ventilation having the highest OR (10.38) compared to PDA (OR = 6.39) and KL-6 (OR = 4.98). Clinical risk factors were assessed using the ROC curve with an area under the curve (AUC) of 0.86 and a sensitivity and specificity of 79% for cutoff value ≥0.34. The finding that PDA is a very strong factor in the development of BPD is consistent with current publications [17, 18]. PDA often leads to pulmonary edema and hemorrhage due to the left-to-right shunt. For this reason, these patients require prolonged mechanical ventilation and oxygen supplementation. The latter two factors may lead to disruption of the lung microvasculature and arrest of alveolar development, resulting in damage to the lung and alveolar structure typical of BPD [19].

It is known that type 2 pneumocyte hyperplasia with fibrotic changes of various degrees occurs in BPD patients [20]. During the regeneration process, type 2 cells strongly express KL-6 antigen. Dilli et al. [21] demonstrated that KL-6/14d levels were significantly higher in infants with BPD (155.2 (15.3–545.6) U/mL) than in infants without BPD (7.9 (7.7–15.6) U/mL) (*P*=0.001). The best predictor was the KL-6 level on postnatal day 14 (area under the ROC curve = 0.88; range: 0.75–1.0; and *P*=0.002). At this time point, the KL-6 level of 59.7 U/mL showed a specificity of 90.0% and a negative predictive value of 85.7% for BPD.

In our binary regression model of KL-6 on day 14, six of the indicators remained in the equation—PDA with the highest OR (23.34), KL-6 (OR = 13.59), GA (OR = 4.58), mechanical ventilation (OR = 4.45), antenatal steroids (OR = 0.19), and sex (for female OR = 0.30). The AUC was 0.91, and sensitivity and accuracy for a cutoff ≥0.37 were 89% and 85%, respectively.

Yamane et al. [22] reported that infants with an earlier gestational age who were mechanically ventilated for a longer time and had impaired lung function had higher KL-6 values. However, there was no significant association between KL-6 levels and static respiratory system compliance (Crs) at any postnatal age. They stated that the serum KL-6 level is not always a reliable marker of the clinical course of BPD. An interesting recent study by Bergantini et al. [23] investigated the urine concentration of KL-6 as a predictive biomarker for the development of BPD in preterm infants. Their results showed that infants with lower gestational age, who are at increased risk for developing BPD, had higher urine concentrations of KL-6 on the first day of life than infants who did not develop BPD. In our study, it is not surprising that infants with lower GA had a much higher likelihood of developing moderate/severe BPD (*P* < 0.001) and that infants with GA ≤ 26 weeks had KL-6 with a significantly higher mean value at both measurement time points.

Antenatal corticosteroids were included in the regression model of KL-6 on day 14. It is well known that in infants born between 23 and 34 weeks GA, antenatal corticosteroid exposure is associated with lower mortality and long-term morbidity (BPD) compared with no exposure [24, 25]. Our study showed a statistically significant difference between the two groups with respect to antenatal corticosteroid prophylaxis (*P*=0.047).

The results of the current study may be limited by the relatively small number of patients from a single center, which may have missed some clinically relevant differences and also leads to wide 95% CI. Another limitation is the old BPD classification used to divide patients into two groups, as new criteria have recently been applied to define disease severity [26]. Third limitation is that KL-6 levels from cord blood/day 1 were not measured because of financial reasons.

## 5. Conclusion

Plasma KL-6 could be a screening biomarker for early detection of infants at higher risk for developing BPD. Markedly elevated levels of KL-6 during the first two weeks of life are seen in infants who develop severe BPD. Regression models are useful tools in medicine to improve the prediction and characterization of specific conditions, which is confirmed by the algorithm from Laughon et al. [14]. The obtained models for predicting the onset of BPD based also on the results of the biomarker KL-6 provide a very good chance of predicting the severity of the disease.

## Figures and Tables

**Table 1:** Perinatal characteristics of the patients.

Characteristics	No/mild BPD (*n* = 48)		Moderate/severe BPD (*n* = 47)		*P*value
	Mean	SD	Mean	SD	
Weight (g)	935.52	160.36	800.64	161.89	<0.001
GA (wks)	26.58	0.77	26.00	0.91	<0.001
Mechanical ventilation (d)	12.96	8.84	34.43	22.34	<0.001
O_2_ therapy (d)	34.00	17.65	54.33	21.74	<0.001
Hospital stay (d)	64.60	14.63	90.55	26.82	<0.001
CPAP, NIV (d)	10.31	5.14	15.42	8.91	0.048

	*n*	%	*n*	%	

Percentage <26 + 0 wks GA	15	27.1	29	57.2	0.077
C-section	33	68.8	34	72.3	0.823
Antenatal steroids	42	87.5	33	70.2	0.047
Maternal hypertension	8	16.7	8	17.0	1.000
Chorioamnionitis, pPROM	28	58.3	24	51.1	0.539
Apgar score <3 at 1 min	13	27.1	16	34.0	0.509
RDS III-IV gr	42	87.5	46	97.9	0.111
Surfactant applied	48	100.0	47	100.0	NA
PDA, lung hemorrhage	2	4.2	13	27.7	0.002
Sepsis	6	12.5	11	23.4	0.190
NEC	1	2.1	6	12.8	0.059
ROP	19	39.6	37	82.2	<0.001

SD, standard deviation; GA, gestational age; NIV, noninvasive ventilation, pPROM, preterm premature rupture of membranes; PDA, persistent ductus arteriosus; RDS, respiratory distress syndrome; NEC, necrotizing enterocolitis; ROP, retinopathy of prematurity.

**Table 2:** Area under the curve (AUC), *P* values, cutoff values, and values of screening test validation criteria for KL-6, mechanical ventilation, GA, weight, model 7d, and model 14d.

Characteristics	AUC	*P* value	Cutoff	Sensitivity	Specificity	Positive predictive value	Negative predictive value	Accuracy
KL-6/7d	0.66	0.007	≥247.5	81	52	62	74	66
KL-6/14d	0.75	<0.001	≥277.5	60	85	80	68	73
MV (days)	0.84	<0.001	≤20	77	81	80	78	79
GA (weeks)	0.69	0.001	≤26.5	66	73	70	69	69
Weight (g)	0.73	<0.001	≤855	66	71	69	68	68
Model 7d	0.86	<0.001	≥0.34	79	79	79	79	79
Model 14d	0.91	<0.001	≥0.37	89	81	82	89	85

MV, mechanical ventilation; GA, gestational age.

**Table 3:** OR and 95% CI of analyzed prognostic factors for severe/moderate BPD based on KL-6/7d.

Independent variables	Comparison	Crude		Multivariate	
		OR (95% CI)		OR (95% CI)	
KL-6/7d (U/ml)	≥247.5/<247.5	4.59	(1.83–11.53)	4.98	(1.54–16.08)
PDA, lung hemorrhage	Yes/No	8.79	(1.86–41.57)	6.39	(0.87–46.74)
Mechanical ventilation (d)	≥20/<20	14.18	(5.27–38.19)	10.38	(3.57–30.14)
GA (weeks)	≤26.5/>26.5	5.22	(2.17–12.54)		
Weight (g)	≤855/>855	4.71	(1.98–11.20)		
Antenatal steroids	Yes/No	0.34	(0.12–0.97)		
Sex	Female/Male	0.81	(0.36–1.81)		
C-section	Yes/No	1.19	(0.49–2.88)		
Complicated pregnancy	Yes/No	0.76	(0.19–3.04)		
Apgar score <3 at 1 min	Yes/No	1.39	(0.58–3.34)		

PDA, persistent ductus arteriosus; GA, gestational age.

**Table 4:** OR and 95% CI of prognostic factors for severe/moderate BPD based on KL-6/14d.

Independent variables	Comparison	Crude		Multivariate	
		OR (95% CI)		OR (95% CI)	
KL-6/14d (U/ml)	≥277.5/<277.5	8.63	(3.20–23.25)	13.59	(3.19–57.96)
PDA, lung hemorrhage	Yes/No	8.79	(1.86–41.57)	23.34	(2.14–254.24)
Mechanical ventilation (d)	≥20/<20	14.18	(5.27–38.19)	4.45	(1.23–16.16)
GA (weeks)	≤26.5/>26.5	5.22	(2.17–12.54)	4.58	(1.16–18.06)
Antenatal steroids	Yes/No	0.34	(0.12–0.97)	0.19	(0.04–0.95)
Sex	Female/Male	0.81	(0.36–1.81)	0.30	(0.08–1.12)
Weight (g)	≤855/>855	4.71	(1.98–11.20)		
C-section	Yes/No	1.19	(0.49–2.88)		
Complicated pregnancy	Yes/No	0.76	(0.19–3.04)		
Apgar score <3 at 1 min	Yes/No	1.39	0.58–3.34		

PDA, persistent ductus arteriosus; GA, gestational age.

## Data Availability

The datasets used and analyzed during the current study are available from the corresponding author upon reasonable request.

## Additional Points

*Key Points*. Plasma K-6 could be a screening biomarker for early detection of infants at increased risk of BPD. Early detection of the most at-risk ELBW infants could lead to early treatment of the disease. This could have a positive impact on public health policy and practice.
